# Visual Working Memory Contents Bias Ambiguous Structure from Motion Perception

**DOI:** 10.1371/journal.pone.0059217

**Published:** 2013-03-19

**Authors:** Lisa Scocchia, Matteo Valsecchi, Karl R. Gegenfurtner, Jochen Triesch

**Affiliations:** 1 Frankfurt Institute for Advanced Studies, Johann Wolfgang Goethe University, Frankfurt am Main, Germany; 2 Giessen University, Department of Psychology, Giessen, Germany; 3 University of Milano-Bicocca, Department of Psychology, Milano, Italy; University of California, Davis, United States of America

## Abstract

The way we perceive the visual world depends crucially on the state of the observer. In the present study we show that what we are holding in working memory (WM) can bias the way we perceive ambiguous structure from motion stimuli. Holding in memory the percept of an unambiguously rotating sphere influenced the perceived direction of motion of an ambiguously rotating sphere presented shortly thereafter. In particular, we found a systematic difference between congruent dominance periods where the perceived direction of the ambiguous stimulus corresponded to the direction of the unambiguous one and incongruent dominance periods. Congruent dominance periods were more frequent when participants memorized the speed of the unambiguous sphere for delayed discrimination than when they performed an immediate judgment on a change in its speed. The analysis of dominance time-course showed that a sustained tendency to perceive the same direction of motion as the prior stimulus emerged only in the WM condition, whereas in the attention condition perceptual dominance dropped to chance levels at the end of the trial. The results are explained in terms of a direct involvement of early visual areas in the active representation of visual motion in WM.

## Introduction

When we interact with our environment, we are often faced with noisy or ambiguous sensory information. Under those conditions, what we perceive can be largely determined by the state of our cognitive system, including our beliefs and expectations. This might be adaptive given that our expectations are broadly consistent with the laws and statistics of the environment. However, our visual perception might also be prone to the influence of more volatile cognitive factors. One prominent and ever-changing aspect of our mental state are the contents of working memory. Visual working memory is the system that underpins our ability to briefly store and actively operate on visual representations. As such, it is fundamental for most activities requiring vision: from learning a new way to the bus station to jotting down the bus schedule in the diary. Indeed, numerous studies have investigated the effects of retaining an item in visual WM on the attentional processing of subsequently presented items [1]–[5]. Recent evidence further showed that a visual search target is not only processed faster, but also more accurately when it is embedded in an object that looks like a memorized object [6], and that coherent motion pulses are more easily identified within a stream of incoherent motion when their direction matches the one of a memorized stimulus [7].

In the present study we ask whether holding a visual object in WM may have a direct impact on the way subsequently presented objects are perceived, particularly when our visual system has to deal with information ambiguous to the point of generating bistable perception.

When viewing a bistable stimulus, the observer perceives it switching spontaneously and unpredictably between two (or more) alternative interpretations. It is well established that bistable perception is prominently influenced by low-level factors, such as neural satiation, neural noise and competition between representations at different levels of the visual pathway. Several models have been proposed to account for spontaneous perceptual alternations solely on the ground of low-level mechanisms [8]–[12]. However, even current low-level accounts of binocular rivalry [13], where ambiguity is induced by displaying incompatible monocular images to the two eyes, leave open the possibility of top-down influences on rivalry dynamics. In other words, contemporary models that posit neural adaptation and noise as necessary factors leading to perceptual alternations, typically consider these factors as susceptible to cognitive modulations. Other models regard bistable perception as the outcome of continuous interaction between lower-level and higher-level brain areas [14]–[16].

The question whether bistable perception is amenable to cognitive influence has a long history [17]. A role of subjective intention, or task instructions, in the perception of ambiguous stimuli is now well established [18]–[20]. Although there is general agreement on the observers’ capability to voluntarily control the alternation rate between two percepts across a wide range of bistable stimuli [21]–[24], the strength of intentional influences on the ability to reverse varies according to the kind of ambiguous stimuli being used: within the category of reversible figures, content-dependent perceptual switches (i.e: when the reversal entails a reconstruction of the meaning, as in the case of Rubin's vase/faces) are more amenable to top-down influence than perspective-dependent switches (i.e: when the reversal entails a reference-frame realignment only, as in the case of the Necker cube) [25]. Likewise, the effect of selective attention on perceptual dominance seems to depend on the type of bistable stimulus. Focusing attention on one of the alternative interpretations increases dominance durations of the attended percept in the case of reversible figures [26]–[28], but not in the case of binocular rivalry [28]. However, endogenous attention can influence dominance durations during rivalry when participants have to track changes in one of the rival stimuli [29]. Furthermore, the deployment of cognitive resources to a secondary task reduces the perceptual alternation rate in the case of reversible figures [30], [20], binocular rivalry [31] and ambiguous apparent motion stimuli [32]. Top-down factors other than intention and attention have also been proposed to influence the perception of ambiguous stimuli, in particular of reversible figures. Among them are knowledge that the figure is reversible [33], [34], prior inspection of the possible alternatives [35]–[38], imagery [38], [39], [20], semantic priming [40], [41], and the motivational state of the observers [42]. Finally, numerous studies in the neurophysiology and neuroimaging domains endorse the view that extra-striate [43]–[45] and even higher, non-visual areas, such as the frontal and parietal cortex [46], [47], underlie perceptual alternations during multistable perception. On this ground, Leopold and Logothetis [48] proposed that perceptual reversals are the epiphenomena of a reorganization of activity throughout the visual cortex that is initiated by central, supra-modal cortical structures.

Studying the effects of visual WM contents on the perception of ambiguous stimuli may provide us with insights on two important issues: the role of cognitive processes in the build-up of our perceptual world and the level at which top-down influence occurs. We tested whether holding in WM unambiguous motion information can affect ambiguous motion perception. As a control, participants focused their attention on the unambiguous stimulus but did not memorize it. This simple paradigm allowed us to directly assess the influence of the cognitive system on what we see: if the WM and the visual systems are independent and perception is exhaustively determined by the input coming from the retina, we should observe the same pattern of results when memorizing and when attending to the unambiguous stimuli. Instead, if perception is permeable to different types of cognitive influence, we may observe a distinct pattern of results in the two conditions. Furthermore, a spillover of WM contents into perception would be consistent with a direct involvement of early sensory areas in the representation of motion in WM [49], [50].

## Methods

### Observers

Twenty-nine naïve observers participated in the Experiment. All observers had normal or corrected to normal vision and were paid for their participation. Participants provided written informed consent in agreement with the Declaration of Helsinki. Methods and procedures were approved by the local ethics committee LEK FB06 at Giessen University.

### Stimuli

We employed ambiguous and unambiguous structure-from-motion (SFM) spheres as stimuli. Each SFM sphere consisted of a two-dimensional projection of a three-dimensional sphere: it was composed of single dots rotating rigidly across its imaginary surface, along the vertical axis, and giving the appearance of a three-dimensional structure. The sphere rotated unambiguously when only the dots moving along the front surface were displayed to the observer, whereas its rotation direction was ambiguous when both the front and the rear surface were displayed. SFM stimuli were presented at the center of a black screen (0.43 cd/m^2^, 1280×1024 pixels, 100 Hz, distance 47 cm) and subtended 14° in diameter. Ambiguous and unambiguous spheres consisted of orthographic projections of 1000 white dots (55.1 cd/m^2^). Each dot had unlimited lifetime and subtended 0.1° in width and height. On each trial, the standard stimulus rotated at 70, 80, 90, 100 or 110°/s. The standard stimulus could be presented randomly in the first interval (as the memory sample) or in the second interval (as the memory test). The comparison stimulus speed was adjusted adaptively via a staircase procedure aiming at 75% of correct responses: after a correct response at the memory test, the velocity difference between standard and comparison stimulus decreased by 1.5%, whereas it increased by 4.5% after an incorrect response. The ambiguous stimulus rotation speed was 45°/s in both experiments.

### Procedure

**Figure 1:**
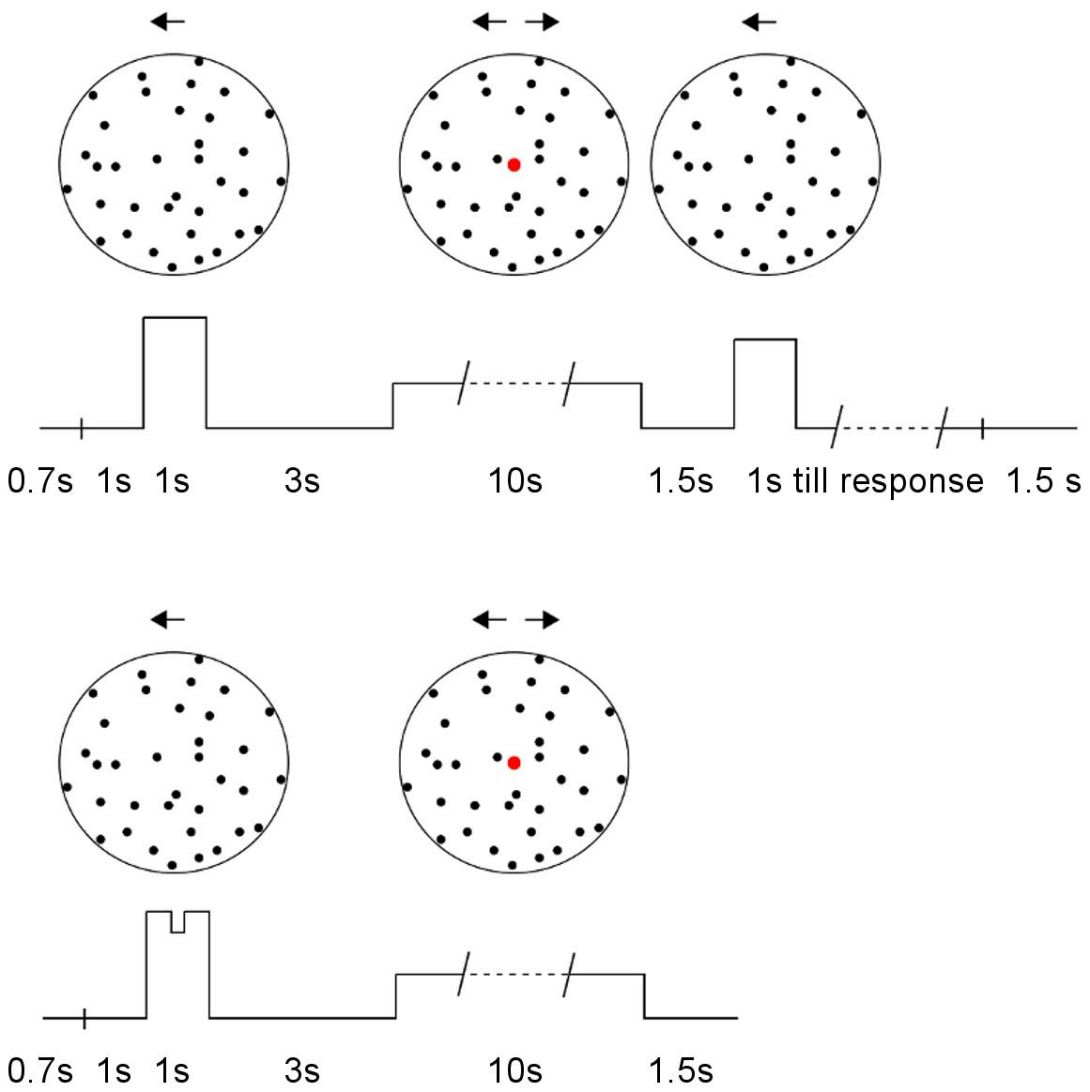
Trial procedure in the WM condition (*upper panel*) and in the attention condition (*lower panel*). The direction of motion of the stimuli is represented by solid arrows above them, their duration and speed is depicted as a line below them. Observers memorized the speed of motion of the initial unambiguous sphere in the WM condition and reported the brief change in its speed in the attention condition. Subsequently, observers continuously reported the perceived direction of motion of the ambiguous sphere. Only in the WM condition observers reported whether the speed of the unambiguous sphere presented at the end of the trial was higher than the memorized one.

All participants performed both a WM and an attention control condition, in a counterbalanced order. As for the WM condition, the procedure is illustrated in Figure 1, top panel: each trial began with a 0.7 s display of the instruction to memorize the subsequently presented stimulus, followed by a blank screen for 1 s. Then a SFM sphere was presented for 1 s, unambiguously rotating either clockwise or anticlockwise with respect to its vertical axis. Afterwards, a red fixation dot (0.2°, 12.6 cd/m^2^) was displayed: it was presented over a blank screen for 3 s and over the center of an ambiguously rotating SFM sphere for 10 s. Then, a blank screen was displayed for 1.5 s, followed by the unambiguous memory test for 1 s, whose motion direction was the same as the memory sample. After 1 s the memory test stopped its motion and was statically displayed on the screen until participants provided their response. Finally, a blank screen was displayed for 1.5 s, before the start of a new trial. Participants were required to maintain fixation whenever the red dot was presented (i.e: during the display of the ambiguously rotating stimulus and the immediately preceding 3 s). The experiment ran on a Dell Precision T390 computer (Dell, Inc., Round rock, Texas) controlled via Matlab and the Psychtoolbox [51]. Stimuli were presented on an Iiyama VisionMaster 22 inch CRT monitor (Iiyama Corporation, Tokyo, Japan). Participants were required to indicate their perceived direction of motion of the ambiguously rotating sphere, by pressing the left or right arrow keys with their right hand. They did not press any key if they did not clearly perceive a unique direction of motion. Participants’ responses were sampled at 100 Hz. As for the memory task, participants were asked to indicate the interval at which the faster stimulus was presented, by pressing 1 or 2 with their left middle or index finger. Participants performed 40 trials overall, 20 for each direction of motion of the unambiguous stimulus. They were allowed to take a break every 5 trials and the whole experimental session took about one hour. Head movements were constrained using a chin-rest.

In the attention control condition, the experimental procedure was the same as in the WM condition, with the following exceptions: first, the instruction to memorize the stimulus was replaced by the instruction to judge whether it increased or decreased its speed. Second, a 200 ms change of speed of the unambiguously rotating stimulus was introduced after 400 ms from its onset: participants pressed the up arrow key to signal an acceleration of the unambiguous stimulus speed and the down arrow key to signal a deceleration. Third and final, no memory test stimulus was displayed and the presentation of the ambiguous stimulus was immediately followed by a 1.5 s blank interval.

### Data Analysis

We sorted data by direction of the unambiguous stimulus: the perception of the ambiguous sphere was defined to be either congruent (when it was perceived to have the same direction of motion) or incongruent (when it was perceived to have the opposite direction of motion) with the unambiguous one.

**Figure 2:**
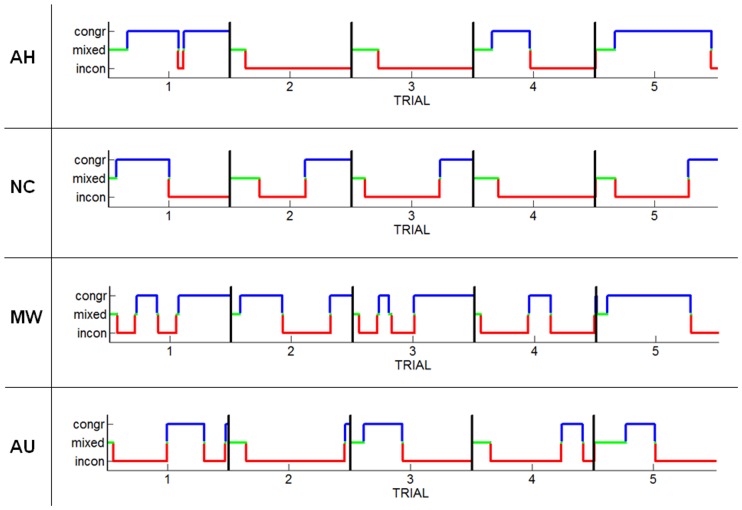
Example of responses from four representative observers during the first 5 trials of the WM condition. Each horizontal *blue* segment represents an episode of perceived congruence between the direction of motion of the ambiguous and the unambiguous sphere, each horizontal *red* segment represents an episode of perceived incongruence. *Green* segments represent mixed dominance. *Black* vertical lines denote the end of a trial.

The display time of the ambiguous stimulus was limited to 10 s, as we wanted to test WM performance after its presentation. For this reason, a consistent number of percepts ended not because of a spontaneous switch to a different percept, but because of the time constraints of stimulus display: on average, 55.4±22.8% of participants’ responses were abruptly truncated as a result of the end of stimulus display. Figure 2 illustrates four representative participants’ responses to the ambiguous stimulus during the first 5 trials of the WM condition. Therefore, percept duration in our experimental manipulation cannot be considered as representative of percept duration under spontaneous viewing conditions. Previous studies that examined dominance durations during ambiguous perception typically allowed for longer viewing periods (one minute or more) and did not take into account truncated percepts [28], [29], [32]. For these reasons, dominance duration was not analyzed in our study. Instead, we focused on the number of episodes of exclusive dominance.

## Results

**Figure 3:**
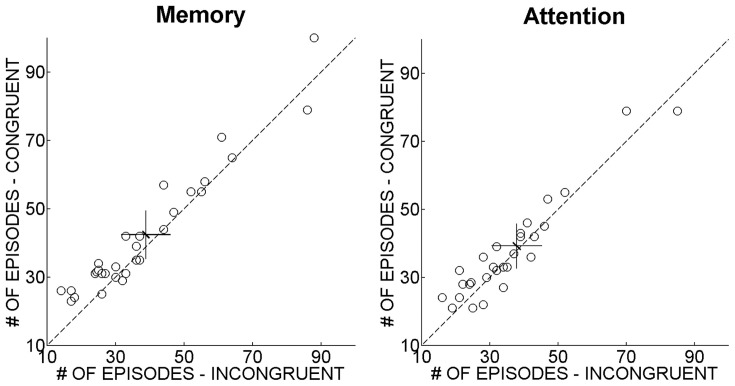
Number of episodes where the reported percept is congruent to the unambiguous stimulus direction as a function of the number of incongruent episodes. The left and the right panels illustrate performance in the WM and in the attention condition, respectively. Each empty circle represents an observer. Vertical and horizontal error bars represent 95% confidence intervals within the congruent and the incongruent condition respectively. Diagonal error bars indicate the between-observers 95% confidence interval of the distance from the bisector. The coordinates of the crossing points of the bars represent the means of the incongruent and congruent conditions, computed across participants. Only in the WM condition the number of congruent episodes was higher than the number of incongruent episodes.

The number of congruent and incongruent episodes across trials was computed separately for each observer in the WM and in the control condition. Figure 3 depicts the experimental results: the left panel represents the number of congruent episodes as a function of incongruent episodes in the WM condition. Participants perceived the direction of motion that matched the memorized one consistently more often than the opposite direction (*t*(28) = 5.04, p<.001). In the attention condition instead, such a difference was not observed (*t*(28) = 1.31, p = .2), as illustrated in the right panel of Figure 3. Importantly, the number of episodes where the direction of motion of the ambiguous sphere was perceived as congruent with that of the unambiguous sphere proved to be greater in the WM than in the attention condition (*t*(28) = 2.81, p<.01).

**Figure 4:**
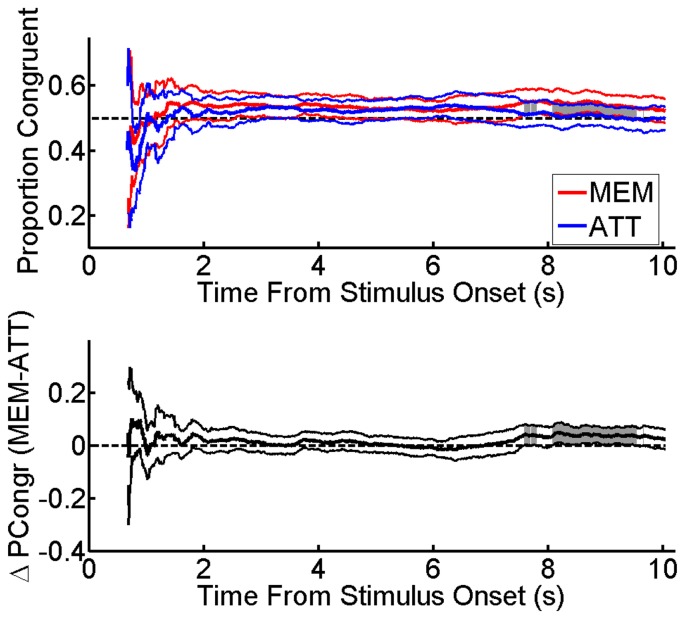
Time-course of dominance. *Upper panel*: average probability of seeing the ambiguous stimulus moving in the same direction as the unambiguous stimulus, expressed as a function of time from the ambiguous stimulus onset. Results are plotted only for time points at which more than 50% of observers provided data. *Red*: WM condition. *Blue*: attention condition. *Lower panel*: the same results are depicted as the difference in instantaneous probability between the WM and the attention condition. Thick and thin lines represent group averages and 95% confidence intervals respectively. Grey insets indicate the time points where the tendency to perceive the congruent motion direction was higher in the WM condition, as evidenced by paired-sample two-tailed t-tests conducted at each time-point on the trial timeline.

A more detailed analysis of participants’ responses was conducted to inspect the time-course of dominance: the results are illustrated in Figure 4. The upper panel depicts the instantaneous probability of seeing the direction of motion that corresponded to the unambiguous stimulus as a function of time from stimulus onset, separately for each experimental condition. The lower panel represents the same results in terms of the difference in instantaneous probability between the WM and the control condition. Data were averaged across participants. The lack of exclusive dominance in the first 670 ms from stimulus onset reflects the fact that the percept took a while to stabilize. Results are plotted only for time points at which more than 50% of observers provided data, yet the response pattern was noisier at the onset of the dominace. Later in the trial, all individual observers provided valid data and the pattern was more stable. To compare the time-course of dominance in the WM and in the attention condition, paired-sample t-tests were performed at each time-point (i.e. every 10 msec). The grey shaded areas in Figure 4 indicate a significant difference at the two-tailed t-test (p<.05). Thick and thin lines represent group averages and 95% confidence intervals, respectively.

**Figure 5:**
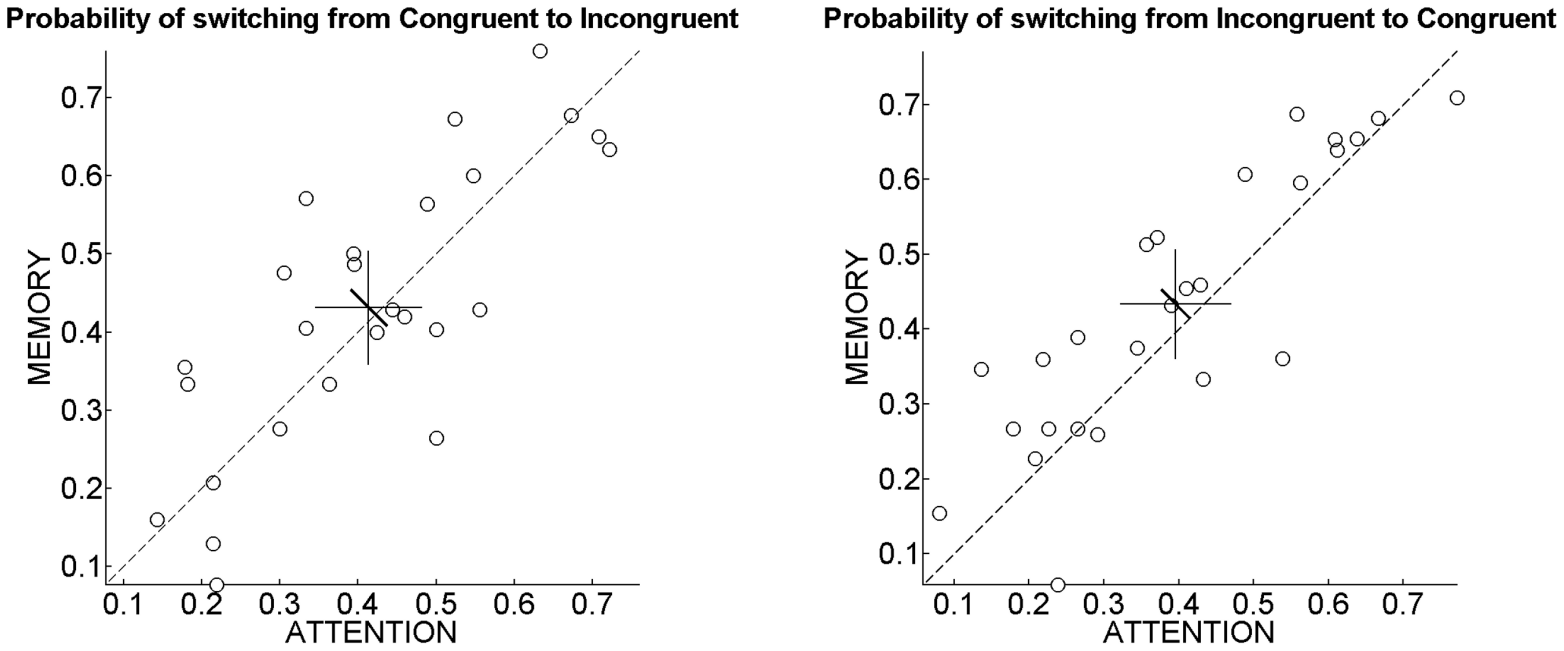
Probability of switching. *Left panel*: Probability of switching from perceiving the same direction of motion as the unambiguous stimulus to perceiving the opposite direction. *Right panel*: Probability of switching from perceiving the opposite direction of motion than the unambiguous stimulus to perceiving the same direction. Probability was computed by diving the number of switches between alternative percepts by the number of episodes across trials, separately for each subject and condition. Performance in the memory condition is plotted as function of the attention condition. Vertical and horizontal error bars represent 95% confidence intervals within the WM and the attention condition respectively. Diagonal error bars indicate the between-observers 95% confidence interval of the distance from the bisector. The coordinates of the crossing points of the bars represent the means of the WM and attention conditions, computed across participants. The probability of switching from an incongruent to a congruent percept was higher in the WM condition as compared to the attention control condition.

Figure 4 clearly shows that, after an initial tendency to perceive the ambiguous sphere as moving in the opposite direction than the unambiguous sphere, average dominance drifted towards the congruent percept in both conditions, but only in the WM condition the congruent percept was dominant until the end of the trial. In the control condition instead, the probability of perceiving the same direction of motion as the unambiguous stimulus dropped to chance levels towards the end of the trial. We therefore further analyzed the data to identify the probability of switching towards the opposite percept in each trial. Each dominance episode within a trial had three possible outcomes: a switch towards the opposite percept, a rebound to the same percept after a period of ambiguous dominance, and the end of the trial. We computed the probability of switching to the opposite percept after removing periods of mixed dominance from the analysis, so that rebound episodes to the same percept where not considered as switches. Given that the number of total congruent episodes was greater in the WM than in the control condition,we calculated the probability of switching to the opposite percept by dividing the number of switches by the number of episodes of the same polarity across trials, separately for each subject and condition (i.e: the number of switches from the congruent to the uncongruent percept was divided by the number of congruent episodes and the number of switches from the incongruent to the congruent percept was divided by the number of incongruent episodes). Three participants were excluded from the analysis as they reported no switches in one or more conditions. The results are illustrated in Figure 5: whereas the probability of switching from a congruent to an incongruent percept was comparable in the two experimental conditions (*t*(25) = 0.78, p = .44), switching from an incongruent to a congruent percept was significantly more likely in the WM than in the attention condition (*t*(25) = 2.05, p<.05). This result points to a sustained influence of WM contents on perception over time.

Consistent with this view, the analyses conducted on the first dominance episode across trials failed to highlight significant differences between the WM and the attention condition: the probability of reporting a congruent direction of motion as the first percept of a trial (i.e: the number of trials where the first episode corresponded to a congruent episode over the total number of trials) was comparable in the two conditions (*t*(28) = 1.18, p = .25). Likewise, the latency of the first congruent dominance episodes across trials was similar in the two conditions (*t*(28) = −0.54, p = .6).

## Discussion

This study shows that the way a SFM bistable stimulus appears to the observer is systematically affected by visual information retained in WM during the viewing period. Concretely, we found that holding a rotating stimulus in WM produces a sustained bias in the perception of an ambiguously rotating sphere, as opposed to merely attending to it.

We tested observers’ performance in two conditions that were matched in terms of stimuli and cognitive demands: we found that dominance periods where the perceived direction of the ambiguous sphere corresponded to the direction of the unambiguous sphere were more frequent when participants memorized its speed for delayed discrimination than when they performed an immediate judgment on a change in its speed. Furthermore, the analysis of the time-course of observers’ percepts showed noisier responses at the beginning of the trial; afterwards participants had a preference for perceiving the same direction of motion as the unambiguous stimulus, regardless of the task. As time progressed within the trial, a sustained tendency to perceive the congruent direction emerged only in the WM condition. The literature indicates that fast adaptation mechanisms may affect initial dominance [52], which is compatible with our results. However, the large inter-subject variability of dominance onset makes it difficult to highlight significant effects in the first two seconds after stimulus onset. Instead, a specific WM influence on perception is clearly detectable over longer periods of time. In particular, participants were more likely to switch from perceiving a direction of motion opposite to that of the prior stimulus to perceiving the same direction when they voluntarily held the prior stimulus in WM.

This sustained effect of WM on ambiguous SFM perception is consistent with recent evidence indicating that visual WM contents can bias orientation discrimination [53] and speed discrimination [54] of normal stimuli, after a delay up to 9 seconds from the display of the memory stimulus. The aforementioned studies [53], [54] showed that, when sensory information is unambiguous, the contents of WM can bias the way we perceive objects presented during the WM retention period in a way that resembles (and potentiates) visual aftereffects. Instead, our study shows that, in case of uncertainty, WM contents influence how the visual system disambiguates ambivalent information. These apparently opposite results could be easily reconciled in the light of recent neuroimaging and neurophysiology evidence [49], [55], [56] suggesting that active maintenance of visual stimuli in WM is mediated by early sensory areas. Here we propose that voluntarily holding a visual object in WM can influence bistable SFM perception via the recruitment of neural networks overlapping with those that underpin perception itself. Sustained activity in early sensory areas would likewise mediate the repulsive effect observed in the case of unambiguous stimuli [53], [54], via saturation of orientation- or motion-selective neurons. Instead, when the sensory input is physically ambiguous, the presence of an unambiguous memory trace at the level of early visual areas would strengthen the signal energy in one direction.

Note that to enhance the signal in one direction it is not necessary for the previously displayed stimulus to be actively memorized. A number of studies (see [57] for a review) have demonstrated that physically ambiguous stimuli that cause perception to alternate between incompatible interpretations when displayed continuously, can be stabilized by interleaving their presentation with blank periods. This points to a form of memory storage across subsequent presentations of the stimuli. This sensory memory biases interpretation in terms of facilitation rather than suppression of the prior stimulus, as long as blank intervals last longer than half a second [58], [59]. In our study, a tendency to perceive the ambiguous sphere moving in the same direction of the unambiguous one was observed both in the WM and in the attention condition soon after dominance onset. However, in the WM condition such facilitation lasted until the ambiguous stimulus was removed from view, while in the attention condition perceptual dominance dropped to chance levels at the end of the trial. This result suggests that the display of the unambiguously rotating sphere likely elicited a spontaneous sensory memory trace in both conditions, but only active maintenance in WM produced long-lasting facilitation effects. A simple and plausible explanation of such a result is that voluntarily holding a visual representation in WM exerts a top-down modulation on the same sensory trace that is automatically elicited by stimulus processing, and prolongs its activation over time. Interestingly, Sterzer and Rees (2008) investigated the neural bases of perceptual stabilization during intermittent presentation of binocular rivalry stimuli (a human face and a grating): they observed activity in the fusiform face area during the blank period and found that it was greater following face than grating dominance, whereas no difference was observed after removal of nonrivalrous stimuli. Furthermore, activity in fronto-parietal regions during the blank period strongly correlated with the tendency of individual observers to stabilize perception [60]. These results suggest that perceptual stabilization may be achieved via a sustained activation of sensory areas that is modulated by feedback input from fronto-parietal cortices. A similar correlation between the tendency to maintain a percept and activity in fronto-parietal regions has also been observed in the case of intermittent presentation of an ambiguously rotating SFM sphere [61]. Furthermore, frontal [62] and parietal [63] areas were found to be active immediately prior to spontaneous perceptual reversal, thus suggesting that they could mediate spontaneous switches in perception during constant physical stimulation. If brain areas traditionally known to be involved in cognitive processes such as working memory and attention [64]–[68] play a role in spontaneous perceptual reorganization of ambiguous sensory information, this is all the more reason they could modulate the activity of lower-level sensory areas when observers are actively engaged in cognitive tasks.

As noted in the introduction, there are now numerous studies showing that bistable perception is amenable to cognitive influence. One such study indicates that endogenously generated activity implied in mental imagery plays a role in resolving binocular rivalry in a way that resembles the effect of faint physical stimulation [39]. Our results provide converging evidence for the effect of voluntary mental activity over ambiguous visual representations. However, the effects of visual WM and imagery on ambiguous perception may not be the same. First, mental images can be elicited by verbal instructions in the absence of any physical visual support [70]–[72] whereas WM involves all-or-none encoding of discrete items and their active maintenance to prevent memory decay [69]. Second, and more importantly, several studies show that visual perception influences imagery but not visual WM [73], [74], thus pointing to different operating mechanisms that interact with perception in different ways.

Our results can also be viewed from the perspective of sustained selective attention. The distinction between WM and sustained selective attention is not clear-cut and it has been claimed that WM encoding and maintenance reflects actively sustained attention to a limited number of visual objects, features or events [75]. Indeed, the concepts of WM and of attentional template largely overlap in the literature: the requirement of holding a visual stimulus in WM for subsequent recall has commonly been employed as an endogenous cue to manipulate spatial attention, which would shift towards the location in space that matched the content of WM [1], [2], [4]. However, there is also evidence that WM and visual attention are not one and the same thing [76], [77]. In particular, WM templates may not automatically trigger attention allocation when this is detrimental to performance [3], [78]. Moreover, a recent study [7] showed that feature-based WM and feature-based attention can individually modulate the perception of motion direction and that their effects can additively combine. A challenging issue for future studies will be to ascertain the interplay of visual WM, sustained attention and feature-based attention in the resolution of the visual ambiguities that we encounter.

In conclusion, our results indicate that the visual and the WM system are not independent: our visual world is not uniquely determined by the information reaching our retinas. Instead, the ever-changing contents of WM can influence what we see.
